# Development of a prediction model for late urinary incontinence, hematuria, pain and voiding frequency among irradiated prostate cancer patients

**DOI:** 10.1371/journal.pone.0197757

**Published:** 2018-07-17

**Authors:** Wouter Schaake, Arjen van der Schaaf, Lisanne V. van Dijk, Alfons C. M. van den Bergh, Johannes A. Langendijk

**Affiliations:** 1 Department of Radiation Oncology, University of Groningen, University Medical Center Groningen, Groningen, The Netherlands; 2 Research group Healthy Ageing, Allied Health Care and Nursing, Hanze University of Applied Sciences, Groningen, The Netherlands; University of Oklahoma Health Sciences Center, UNITED STATES

## Abstract

**Background and purpose:**

Incontinence, hematuria, voiding frequency and pain during voiding are possible side effects of radiotherapy among patients treated for prostate cancer. The objective of this study was to develop multivariable NTCP models for these side effects.

**Material and methods:**

This prospective cohort study was composed of 243 patients with localized or locally advanced prostate cancer (stage T1-3). Genito-urinary (GU) toxicity was assessed using a standardized follow-up program. The GU toxicity endpoints were scored using the Common Terminology Criteria for Adverse Events version 3.0 (CTCAE 3.0) scoring system. The full bladder and different anatomical subregions within the bladder were delineated. A least absolute shrinkage and selection operator (LASSO) logistic regression analysis was used to analyze dose volume effects on the four individual endpoints.

**Results:**

In the univariable analysis, urinary incontinence was significantly associated with dose distributions in the trigone (V55-V75, mean). Hematuria was significantly associated with the bladder wall dose (V40-V75, mean), bladder dose (V70-V75), cardiovascular disease and anticoagulants use. Pain during urinating was associated with the dose to the trigone (V50-V75, mean) and with trans transurethral resection of the prostate (TURP). In the final multivariable model urinary incontinence was associated with the mean dose of the trigone. Hematuria was associated with bladder wall dose (V75) and cardiovascular disease, while pain during urinating was associated with trigone dose (V75) and TURP. No significant associations were found for increase in voiding frequency.

**Conclusions:**

Radiation-induced urinary side effects are associated with dose distributions to different organs as risk. Given the dose effect relationships found, decreasing the dose to the trigone and bladder wall may reduce the incidence of incontinence, pain during voiding and hematuria, respectively.

## Introduction

The introduction of intensity modulated radiotherapy (IMRT) and dose escalation has resulted in increased biochemical relapse free survival for localized prostate cancer [1]. Despite this increase in tumor control, adjacent organs at risk (OAR) are exposed to high doses that may lead to increased rates of radiation-induced side effects that have an impact on quality of life [2].

To achieve a reduction in side effect rates by adjusting radiotherapy planning, knowledge of the association between complication risk and dose distribution parameters is required.

Traditionally, a distinction is made between gastrointestinal (GI) and genitourinary (GU) side effects. Dose to specific substructures within and around the anorectum are associated with specific gastrointestinal side effects [3]. Previous studies on genitourinary side effects showed the dose to the bladder was associated with urinary toxicity among patients treated with external beam radiotherapy [4] [5]. Interestingly, dose to the trigone was associated with genitourinary side effects among patients treated with brachytherapy [6,7].

In the vast majority of studies on GU side effects after radiotherapy, toxicity is typically scored as a single cumulative endpoint for all GU side effects taken together, rather than a single score for incontinence, hematuria, pain and increased voiding frequency individually. Presently no multivariable models exists on dose to regions within the bladder and specific late side effects among IMRT treated prostate cancer patients. Therefore, the main objective of this study was to develop multivariable NTCP (Normal Tissue Complication Probability) models for urinary incontinence, hematuria, pain and increased voiding frequency taking into account dose distributions to the bladder as a whole, several regions within the bladder and other candidate prognostic factors.

## Materials and methods

### Patients

This prospective cohort study was previously described in [3] and was composed of 243 patients with prostate cancer confined to the prostatic capsule (stage T1-3). All patients were treated with radiotherapy between 2005 and 2009. The minimal follow up of patients alive was 3 years. Radiotherapy was delivered using linear accelerators with 6 MV photons by intensity modulated radiotherapy (IMRT). Patients were treated 5 times a week to a total dose of 78 Gy on the planning target volume (PTV), using 2 Gy per fraction. In the current patient cohort, no pelvic lymph nodes were irradiated as part of the treatment. Setup accuracy was verified during delivery by matching bony anatomy or implanted fiducial markers. Most patients with locally advanced prostate cancer received adjuvant hormonal treatment (Table 1). Diabetes was defined as “use of hypoglycemic drugs”, smoking was defined as “any kind of smoking history”. These patient characteristics were retrospectively assessed from detailed patient charts. For the purpose of the current analysis, only patients biochemically failure free at three years after treatment were eligible for this study.

**Table 1:** Patient and treatment characteristics.

		Number of patients	%
Age			
	≤ 70 years	139	57
	>70 years	104	43
Tumor classification			
	T1	92	38
	T2	107	44
	T3	44	18
PSA			
	< 4	8	4
	4–10	75	30
	>10	160	66
Gleason			
	5–6	87	36
	7	97	40
	8–10	59	24
Treatment related factors			
	Adjuvant hormonal therapy	104	43
	Fiducial markers	73	30
Pre-treatment related factors			
	History of diabetes	29	12
	Smoking	77	32
	History of cardiovascular disease	82	34
	History of abdominal surgery	90	37
	Anticoagulants use	63	26
	TURP	52	21

PSA: Prostate-specific antigen

TURP:transurethral resection of the prostate

### Ethics statement

All patients were subjected to a prospective data registration program in which complications and treatment results in terms of local control and survival are prospectively assessed. This is done within the framework of routine clinical practice in which outcome and complications are systemically scored as part of a quality assurance program. All data obtained and used for this study has been anonymized.

The (Dutch) Medical Research Involving Human Subjects Act is not applicable to data collection as part of routine clinical practice and use of these data for scientific papers regarding the quality assurance program. Only research that is within the scope of the Medical Research Involving Human Subjects Act needs approval from an (accredited) ethics committee. Therefore, the hospital ethics committee (the Medisch Ethische Toetsingscommissie; METc) concluded that data collection by this program is regarded as part of routine patient care and granted us a waiver from needing ethical approval for the conduct of this study.

In the Netherlands a patient of course has to give his/her consent for the collection of the extra data on behalf of the quality assurance program and the use of these data for scientific papers regarding the quality assurance program. However, according to Dutch legislation, consent is free of form, and verbal consent is sufficient. Therefore, patients were asked to participate in this quality assurance program and asked for permission to use their data for the program and scientific papers regarding the program. Refusal of participation was recorded in their medical record.

### Target and organ at risk delineation

Three Planning Target Volumes (PTV) were defined: the PTV46 included the prostate and vesicles, the PTV70 included the prostate and the basis of the vesicles and the PTV78 included the prostate only.

**Fig 1:**
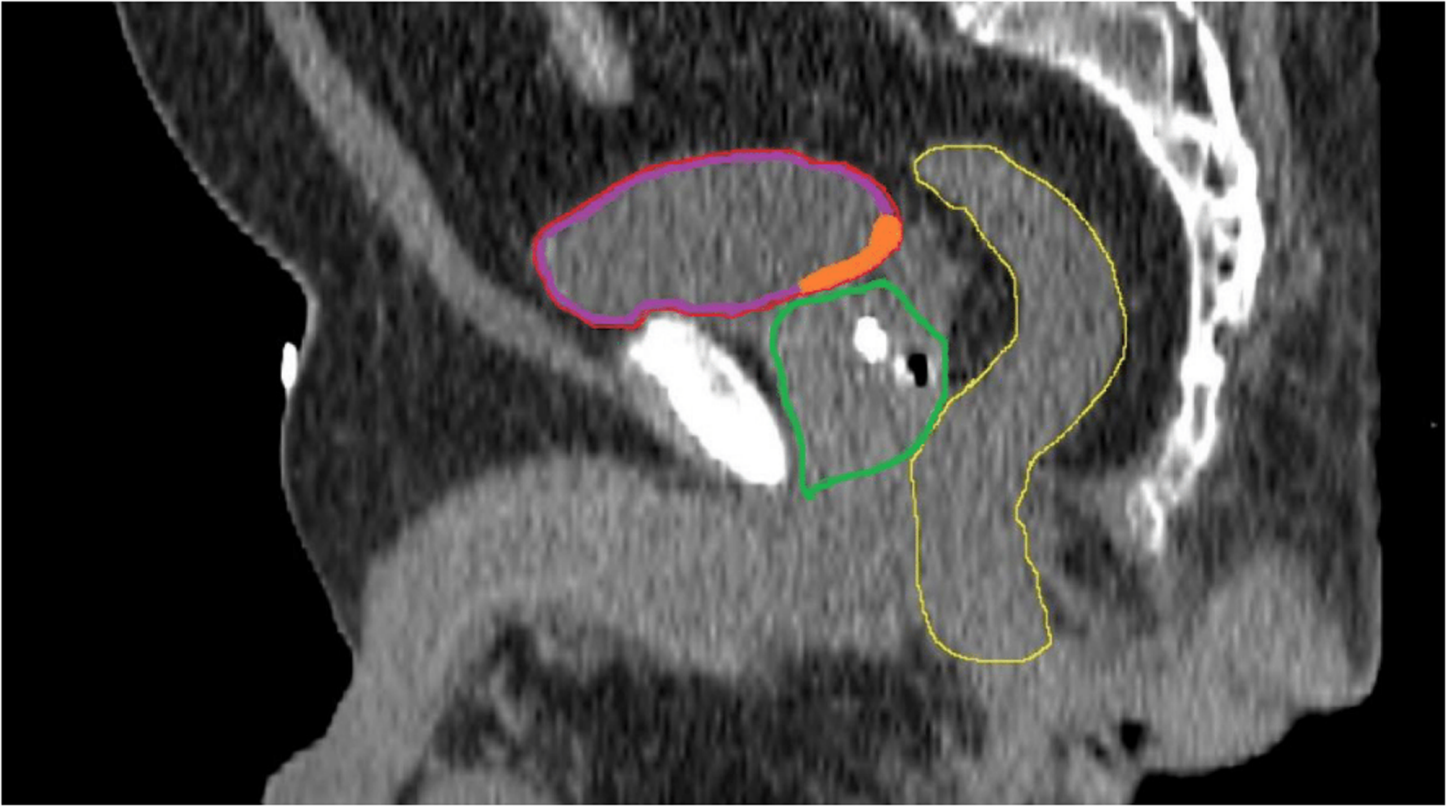
Sagittal view of the bladder (red), bladderwall (purple), trigone (orange), prostate (green), anorectum (yellow).

The full bladder was delineated as part of the treatment planning. Patients were instructed to urinate and drink half a liter of water one hour prior to radiotherapy. The bladder wall was created using an inner ring within the full bladder of 3.3mm [8]. The trigone was defined as the triangle-shaped structure between the transition of the ureters in the bladder wall cranially and the transition of the urethra into the bladder wall caudally [9] (Fig 1).

### Endpoints

Side effects were assessed prospectively using questionnaires filled out by patients treated between 2005 and 2009. The questionnaires have been previously used in a multicenter randomized phase III trial [10] and at our institute [11]. Using these questionnaires, the different endpoints were scored according to the Common Terminology Criteria for Adverse Events version 3.0 (CTCAE 3.0) scoring system [12]. Late toxicity was assessed at six months, 12 months, 24 months and 36 months after treatment, finally resulting in a single maximum toxicity score per endpoint over the entire three years. The minimal follow up of patients alive was 3 years.

Urinary incontinence grade ≥ 2 was defined as spontaneous loss of urine and when use of pads was indicated. Hematuria grade 1 was defined as minimal bleeding when no intervention was indicated, while hematuria grade 2 was defined as bleeding requiring medical intervention. Pain or discomfort during urinating grade 1 was defined as mild pain not interfering with function, while pain grade ≥2 was defined as pain interfering with instrumental activities of daily living (ADL). Finally, voiding frequency increase was defined as an increase of >2 times normal (grade ≥2).

### Statistical analysis

The candidate prognostic factors of the four endpoints in our analysis were selected based on available literature on GU-NTCP modeling [4–6] and included the mean dose for each organ at risk and the relative volumes receiving 5–70 Gy, in 5 Gy bins (V5-V70). Additionally, we included age, adjuvant hormonal treatment, and pre-treatment factors (Table 1) as candidate predictors, which were retrieved retrospectively from the patient charts.

To show the crude effect of each endpoint, a univariable logistic regression analysis was performed on every endpoint. For the development of the multivariable prediction models the least absolute shrinkage and selection operator (LASSO) method in R was used, available in the Lasso and Elastic-Net Regularized Generalized Linear Model package, version 2.0–2 [13]. This is a logistic regression analysis with a penalty for the magnitude of the regression coefficients to prevent overfitting [14]. Because of the high collinearity of the candidate predictors, the set of variables was reduced prior to the LASSO analysis; from the dose variables that had an intercorrelation > 0.80 only the most significant predictor (as measured by the p-value in univariable analysis) remained a candidate prognostic factor.

Subsequently, the variables selected by LASSO were fitted again to the data with logistic regression and internally validated through bootstrapping. This validation gives a measure of optimism of the model, which can be used to correct the coefficients of the model performance accordingly. Model performance was described using various validation measures [15] [16]. The discriminating ability of the model was described by the Area Under the receiver operating characteristic Curve (AUC). Nagelkerke’s R^2^ was calculated as a pseudo measure of explained variance. The gain and intercept of the model calibration were calculated, and the calibration was evaluated using a Hosmer-Lemeshow test [17]. Results with p<0.05 were considered as significant.

Finally, for each endpoint a NTCP curve was constructed based on the corrected regression coefficients from then internal validation (Eq 1).

$$\text{NTCP}=\frac{1}{\left(1+e^{-S}\right)}$$ (1)

## Results

### Urinary incontinence

Twenty nine out of 243 patients (12.0%) experienced grade 2 or higher urinary incontinence. The candidate predictors of urinary incontinence included dosimetric predictors of all bladder structures and pre-treatment variables diabetes, age, cardiovascular disease, abdominal surgery, adjuvant hormonal treatment and TURP. Nine patients experienced grade ≥ 2 incontinence prior to radiotherapy. As these patients already experienced the endpoint of the model before treatment, they were excluded from the multivariable model on incontinence. In the univariable analysis and the V55-V75 and mean dose of the trigone were associated with urinary incontinence (Table 2).

**Table 2:** Univariable logistic regression analysis for urinary incontinence, hematuria and pain during voiding. Only p-values < 0.05 are shown.

		Odds ratio (OR)*	CI	p-Value
*Urinary Incontinence* ≥ *grade 2 (n = 20)*				
Trigone	Mean dose	1.11	1.02–1.20	0.015
	V55	1.08	1.01–1.16	0.027
	V60	1.06	1.01–1.11	0.016
	V65	1.05	1.01–1.08	0.010
	V70	1.03	1.01–1.05	0.008
	V75	1.01	1.00–1.02	0.042
*Hematuria* ≥*1 (n = 23)*				
Cardiovascular disease		2.845	1.19–6.80	0.019
Anticoagulants use		2.424	1.01–5.85	0.049
Bladderwall	Mean dose	1.028	1.00–1.06	0.032
	V40	1.017	1.00–1.03	0.040
	V45	1.019	1.00–1.04	0.024
	V50	1.020	1.00–1.04	0.015
	V55	1.021	1.00–1.04	0.014
	V60	1.022	1.01–1.04	0.011
	V65	1.024	1.01–1.04	0.007
	V70	1.026	1.01–1.04	0.004
	V75	1.027	1.01–1.04	0.002
Bladder	V70	1.015	1.00–1.03	0.029
	V75	1.015	1.00–1.03	0.021
*Pain during voiding*				
TURP		2.46	1.01–5.99	0.048
Trigone	Mean dose	1.106	1.03–1.19	0.008
	V50	1.048	1.00–1.10	0.043
	V55	1.047	1.01–1.09	0.024
	V60	1.042	1.01–1.08	0.015
	V65	1.038	1.01–1.07	0.008
	V70	1.030	1.01–1.05	0.003
	V75	1.021	1.01–1.03	0.001

For dose variables OR: increase per 1 Gy increase in dose, for volume parameters: increase per 1% increase in volume

TURP: transurethral resection of the prostate

**Fig 2:**
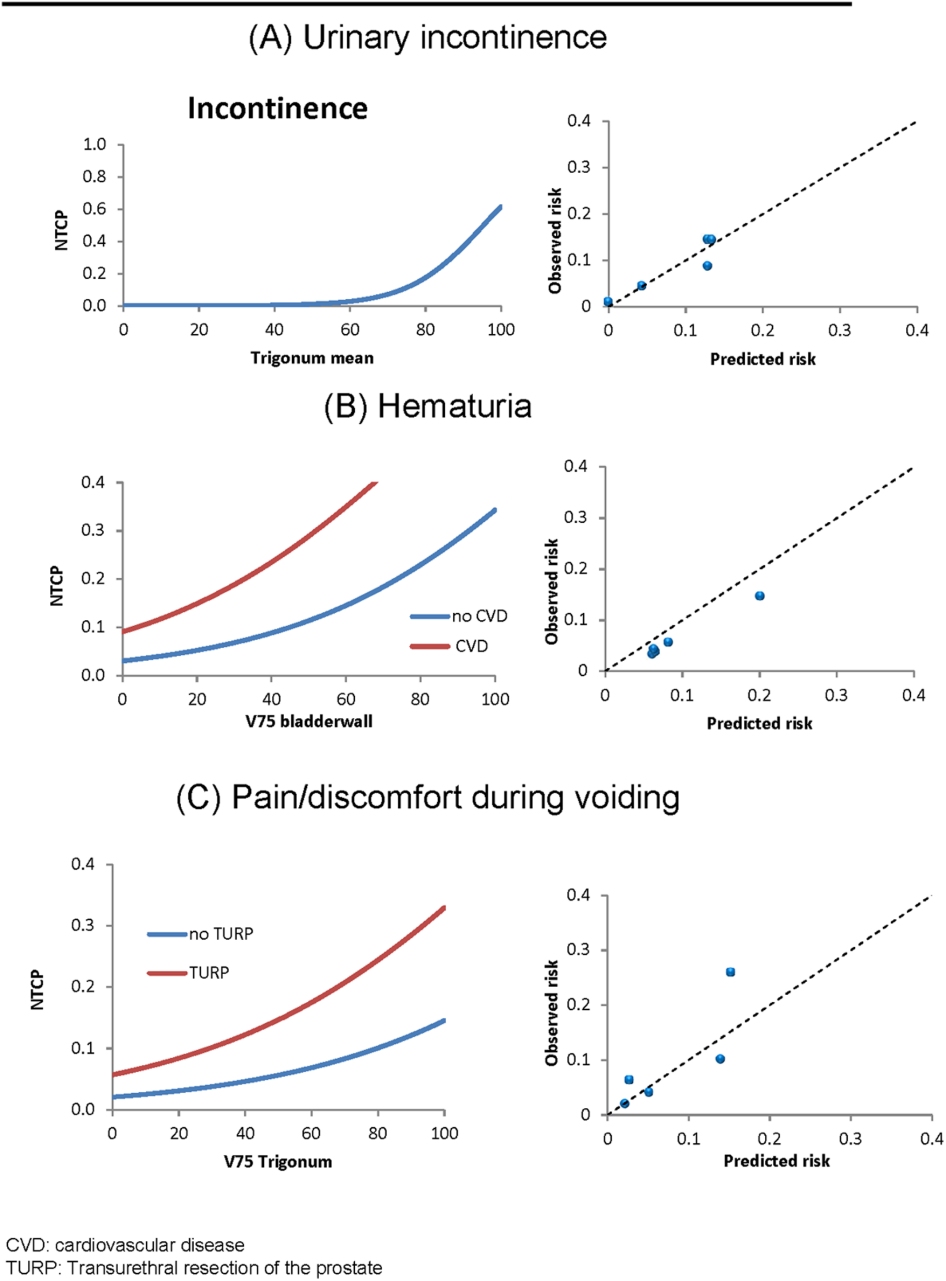
Final logistic regression analysis for urinary incontinence, hematuria and pain/discomfort during voiding. The left graphs represent relative volumes with corresponding NTCP risk. The right graphs represent calibration plots for internal validation. The blue points represent the Hosmer–Lemeshow groups and the dashed line represents the identity line.

The final multivariable analysis resulted in a model with one predictor (Fig 2), i.e. the trigone mean dose (Confidence Interval (CI) Odds Ratio (OR): 1.02–1.20), with a corrected AUC of 0.66 (CI: 0.58–0.78) and a corrected R-square of 0.10 (Table 3).

**Table 3:** Performance and calibration measures for the multivariable model for urinary incontinence, hematuria and pain during voiding. Apparent measures were calculated using the complete dataset on which the model was trained; the corrected measures were adjusted for optimism as calculated with a bootstrapping procedure.

Performance and calibration measure	Urinary incontinence		Hematuria		Pain during voiding	
	Apparent	Corrected	Apparent	Corrected	Apparent	Corrected
AUC*	0.66	0.66	0.72	0.71	0.77	0.76
Nagelkerkes R^2^	0.11	0.10	0.13	0.10	0.16	0.13
Discrimination Slope	0.04	0.04	0.08	0.08	0.08	0.08

*AUC: Area under the Curve.

In individual cases, the risk of urinary incontinence can be estimated using formula 1, where S is defined as:

S=-9.67+0.1015∙(trigone(mean))

The Hosmer-Lemeshow test was not significant (chi square of 4.91.71; degrees of freedom (df) = 8; p = 0.77), indicating good agreement between expected and observed complication rates.

### Hematuria

In total, 23 out of 243 (9.5%) patients experienced grade ≥ 1 hematuria, of which 3 (1%) grade 2. Because of the low number of patients with grade 2 toxicity (3), we decided to use grade ≥ 1 hematuria as primary endpoint for this analysis. The candidate predictors for hematuria included dosimetric predictors of all bladder (sub)structures and other pre-treatment variables, including diabetes, age, cardiovascular disease, abdominal surgery, adjuvant hormonal treatment, anticoagulants and TURP.

In the univariable analysis grade ≥ 1 hematuria was associated with the V40-V75 and mean dose of the bladder wall and the V70-V75 of the bladder (Table 2). In addition, significant associations were found with cardiovascular disease and anticoagulant use.

The final multivariable analysis resulted in a model with two predictors (Fig 2), including the bladder wall V75 (CI OR 1.03–1.03) and cardiovascular disease (CI 3.12–3.31), with a corrected AUC of 0.71 (CI: 0.62–0.84) and a corrected R-square of 0.10 (Table 3). In individual cases, the risk of hematuria can be estimated using formula 1, where S is defined as:

S=−3.45+0.028⋅(bladderwall(V75))+1.15⋅(cardiovasculardisease))

With bladder wall(V75) in relative volume % and cardiovascular disease is 1 (yes) or 0 (no).

The Hosmer-Lemeshow test had a chi square of 4.96 (df = 8, p = 0.76), indicating good agreement between expected and observed complication rates.

### Pain or discomfort during voiding

A total number of 24 out of 243 patients (9.9%) experienced moderate discomfort or pain during voiding (≥ grade 2). The candidate predictors of pain or discomfort during voiding included dosimetric predictors of all bladder (sub)structures and a number of other pre-treatment variables including diabetes, age, cardiovascular disease, abdominal surgery, adjuvant hormonal treatment and TURP.

In the univariable analysis the V50-V75, mean dose of the trigone and TURP were significantly associated with increased pain or discomfort during voiding (Table 2).

The final multivariable analysis resulted in a model with two predictors (Fig 2), including TURP (CI OR 1.18–7.83) and trigone (V75) (CI OR 1.01–1.04), with a corrected AUC of 0.76 (CI: 0.67–0.86) and a corrected R-square of 0.13 (Table 3). In individual cases, the risk of pain or discomfort during voiding can be estimated using formula 1, where S is defined as:

S=−3.87+0.021⋅(trigone(V75)+1.06⋅(TURP)

With trigone(V75) in relative volume % and TURP is 1 (yes) or 0 (no).

The Hosmer-Lemeshow test was not significant (chi square 7.84; df 8; p = 0.48), indicating good agreement between expected and observed complication rates.

### Increase in voiding frequency (night and day)

In the univariable and multivariable analysis no significant associations were found for any of the dosimetric or pre-treatment predictors with voiding frequency.

## Discussion

The main objective of this study was to develop multivariable NTCP models for urinary incontinence, hematuria, pain or discomfort during voiding and increase in voiding frequency based on different subregions within the bladder. Urinary incontinence was best predicted by the trigone mean dose. Hematuria was best predicted by the bladder wall V75 and cardiovascular disease. Pain or discomfort during voiding was best predicted by the trigone V75 and cardiovascular disease. No associations were found for increase in voiding frequency.

Our data show that the dose to the trigone may have an impact on the occurrence of GU complaints, which is in line with research on patients with high-dose IMRT (86.4 Gy) [9] and with brachytherapy [6,7]. In both studies complaints were scored using the IPSS (International Prostate Symptom Score (IPSS)), resulting in a single (side effect) score for each patient. As different GU side effects have a different pathophysiology [18], relating each side effect individually to different dose parameters may be more appropriate and results in more appropriate associations between dose-volume parameters and specific side effects.

Urinary incontinence may originate in the trigone of the bladder, as the lower part contains the involuntary internal or pre-prostatic sphincter [18]. A decrease in dose to this region may likely decrease the incidence of urinary incontinence. However, the external sphincter of the bladder may also play a role in urinary incontinence as it controls the voluntary control of voiding. The external sphincter lies directly beneath the prostate and thereby receives high doses. As this structure is hard to distinguish on CT images we were not able to investigate the impact of dose to this region. The use of MRI in delineating target organs may offer better opportunities to analyze the effect of external sphincter dose-volume parameters on incontinence, as the external sphincter can be better identified with MRI [18].

External beam radiotherapy can cause hematuria [19–20], which is most likely related to the high dose areas within the bladder [21]. In the latter study the volume of the whole bladder receiving ≥ 75 Gy was the best predictor for hematuria. Although the univariable analysis of the present study showed similar results, no such relationship was found in the final multivariable model, which actually showed that the dose to the bladder wall was a better predictor of hematuria than the entire bladder dose. These results suggest that the dose to the entire bladder is a surrogate for the importance of the dose to the bladder wall. From a pathophysiological point of view, it seems evident that dose hotspots to the bladder wall are more indicative of side effects [22–23]. Anticoagulants use was significantly related to hematuria in the univariable analysis, which is in accordance with a study by Palorini [24]. In that study, cardiovascular drugs were found as risk factors for a decreased IPSS score, indicating a possible impaired healing process of radiation-induced damage in patients with microangiopathic disease. Interestingly, anticoagulants were not significant anymore in our multivariable model. This may be caused by the fact that a significant number of patients with cardiovascular disease used anticoagulants. Cardiovascular disease NTCP models performed better than NTCP models with anticoagulants, indicating the importance of taking into account cardiovascular disease in treatment of prostate cancer patients [25–26].

The question may arise what the impact is of volume and motion of the bladder on finding dose-volume relations. A study by Palorini [27] on bladder motility during treatment showed that the cranial and anterior part of the bladder exhibits large systematic variation. The caudal part of the bladder however is relatively independent of bladder filling and may therefore be a reliable predictor in optimizing prostate radiotherapy. The trigone is a relatively rigid part of the bladder, as the bladder neck is surrounded by the prostate and encompassing puboprostatic ligaments [28]. A study on prostatectomy shows that sparing of these ligaments may result in less urinary incontinence [29]. Taking these results into account, the dose to the trigone that we found to be predictive of urinary incontinence and pain, may be a surrogate for these ligaments. However, these ligaments were not visible on CT and were therefore not investigated as an organ at risk for any of the four endpoints.

Although increase in voiding frequency is reported frequently as a side effect resulting from prostate radiotherapy, we were not able to find an association between dose to different anatomical regions and increase in voiding frequency. In a recent study using a pixel-wise analysis of dose-surface maps [30], a relation was found between the dose to the posterior bladder at 5–12 mm from the base and an acute increase in voiding frequency. Therefore, future research should focus on this endpoint to confirm this dose-volume relation in order to prevent a decrease in quality of life of prostate cancer patients.

A limitation of this study was the low number of events for each endpoint. The internal validation showed that for these data the models performed relatively well and despite the low number of events the robustness in this dataset was relatively large [3] [31]. There are different ways to analyze dose-effect relationships, e.g. LASSO, bootstrapping and “simple” backward regression analysis. To check the robustness of our analysis, all of the aforementioned procedures were tested in our data and all led to more or less the same results, indicating our results were relatively independent of the type of analysis that was used and relatively independent of the low number of events. However, external validation in other datasets is warranted. We are currently working on a study to validate our data externally as was done at our institute for head and neck cancer patients [32].

Another limitation of this study is the variability of the bladder volume during treatment. Research has shown that patient motion, bladder centroid motion and bladder filling may have an impact on pretreatment imaging [33]. We recommend researchers to take this into account in future studies on prostate NTCP-modeling by using daily imaging techniques and deformable image registration.

This study shows that different anatomical subregions within the bladder are related to different side effects. Urinary incontinence and pain during voiding is related to dose to the trigone and hematuria is related to the dose in the bladder wall. This information can be used in treatment plan optimization. Additional prospective studies are needed to confirm that dose reductions to these regions result in less side effects.
